# A Seed Expansion Graph Clustering Method for Protein Complexes Detection in Protein Interaction Networks

**DOI:** 10.3390/molecules22122179

**Published:** 2017-12-08

**Authors:** Jie Wang, Wenping Zheng, Yuhua Qian, Jiye Liang

**Affiliations:** Key Laboratory of Computational Intelligence and Chinese Information Processing of Ministry of Education, School of Computer and Information Technology, Shanxi University, Taiyuan 030006, Shanxi, China; xhcwj@sina.com (J.W.); wpzheng@sxu.edu.cn (W.Z.); jinchengqyh@126.com (Y.Q.)

**Keywords:** graph clustering, protein complex detection, seed expansion, protein interaction network

## Abstract

Most proteins perform their biological functions while interacting as complexes. The detection of protein complexes is an important task not only for understanding the relationship between functions and structures of biological network, but also for predicting the function of unknown proteins. We present a new nodal metric by integrating its local topological information. The metric reflects its representability in a larger local neighborhood to a cluster of a protein interaction (PPI) network. Based on the metric, we propose a seed-expansion graph clustering algorithm (SEGC) for protein complexes detection in PPI networks. A roulette wheel strategy is used in the selection of the seed to enhance the diversity of clustering. For a candidate node *u*, we define its closeness to a cluster *C*, denoted as *NC*(*u*, *C*), by combing the density of a cluster *C* and the connection between a node *u* and *C*. In SEGC, a cluster which initially consists of only a seed node, is extended by adding nodes recursively from its neighbors according to the closeness, until all neighbors fail the process of expansion. We compare the *F*-measure and accuracy of the proposed SEGC algorithm with other algorithms on *Saccharomyces cerevisiae* protein interaction networks. The experimental results show that SEGC outperforms other algorithms under full coverage.

## 1. Introduction

In the proteomics era, various high throughput experimental techniques and computational methods have produced enormous protein interactions data [1], which have contributed to predict protein function [2,3] and detect protein complexes from protein–protein interaction (PPI) networks [4]. Prediction of protein complexes can help to understand principles of cellular organization and biological functions of proteins [5,6,7]. A PPI network can be modeled as an undirected graph, where nodes represent proteins and edges represent interactions between proteins. Proteins usually interact with others as a complex to perform their biological functions in cells, such as DNA replication, transcription and protein degradation [8,9,10], so protein complexes are usually dense subgraphs in PPI networks.

Graph clustering [11] is an unsupervised learning technique that groups the nodes of the graph into clusters taking into consideration the edge structure of the graph in such a way that there should be many edges within each cluster and relatively few between the clusters. Clusters in a PPI network are highly interconnected, or dense regions that may represent complexes. Thus, identifying protein complexes is similar to finding clusters in a graph. Various graph clustering algorithms have been developed to identify protein complexes using the information encoded in the network topology. In general, these methods can be classified into two types: Global method and local method, according to whether they produce clusters based on whole view or partial view of graph topology.

Global approaches exploit the global structure information of networks. Girvan and Newman proposed the Girvan and Newman (GN) algorithm [12] to partition network by iteratively removing the edges with highest edge betweeness. Markov clustering algorithm (MCL) [13,14] starts from an initial flow matrix to identify complexes by simulating stochastic flows between nodes in PPI networks. Spectral clustering methods [15] construct a similarity graph from initial PPI network, and then determine clusters based on spectral analysis of the similarity graph. Most global methods partition networks into non-overlapping subgraphs and assign all nodes in a subgraph into a cluster. These methods enable identification of all relevant modules within a PPI network, so they might obtain robust and effective performance for protein complex detection. However, global methods are computationally expensive and limited to relatively small PPI networks [16].

Local clustering methods identify protein complexes by considering local neighbor information in PPI networks instead of global information. A simple strategy of the local method is to enumerate all highly connected subgraphs in PPI networks with density exceeding a specified threshold. Clique Percolation Method (CPM) [17] finds *k*-clique-communities as a union of all *k*-cliques that can be reached from each other through a series of adjacent k-cliques. CFinder method [18] implements this approach and is currently being used in complex detection in PPI networks. Clustering-based on Maximal Cliques (CMC) [19] identifies maximal cliques as candidate clusters and then adds a post processing on highly overlapping cliques to generate final clusters. However, since searching all maximal cliques in a network is an NP hard problem, these algorithms are computationally expensive. Furthermore, these algorithms cannot provide satisfactory coverage. To improve computational efficiency, algorithms utilizing local expansion and optimization are proposed and often classified as “greedy” and “graph growing” algorithms [20]. Most of these algorithms start by selecting a highly ranked node as a seed and then expand the seed to a densely connected group of nodes relying on a local benefit function. Researchers often call these kinds of algorithms “seed expansion methods”. The Molecular Complex Detection (MCODE) algorithm [21] is one of the most classical seed expansion computational methods that can identify densely connected clusters in PPI networks. It first weights all nodes by their *k*-core neighborhood density as local network density, and then expands from highest weighted node by adding nodes whose vertex weight percentage (VWP, weight percentage away from the weight of the seed vertex) is above a given threshold. The weighting scheme of MCODE boosts the weight of densely connected nodes. For a node *v*, MCODE computes the VWP value of *v* to check whether *v* is part of the cluster being considered. The VWP value of a node reflects its relative neighborhood density respective to that of the seed in current cluster. However, VWP value might not be an exact representation to measure the closeness between a node and the current cluster.

DPClus algorithm [22] defines “cluster periphery” of a node with respect to a cluster to address the aforementioned issue. DPClus first weighs an edge by the number of common neighbors between two ends of the edge, and then weighs a node as the sum of the weights of edges incident to the node. For node *v*, its “periphery” respect to a cluster *C* is defined as the fraction of the number of nodes in *C* adjacent to *v* and average link number of node in *C*. However, “periphery” value only considers the connections between node *v* and cluster *C*, without taking into account the neighborhood density information of the node *v* itself.

It first chooses node with the highest weighted degree as a seed that forms an initial cluster. The weight degree of a node is the sum of all of its adjacent edges’ weights, where an edge weight is measured by the number of common neighbors of interacted proteins. The node weight reflects local density in the node’s immediate neighborhood by the number of triangles on it. Then, DPClus iteratively augments the initial cluster by adding nodes if the density and cluster property of the cluster are higher than user-defined thresholds.

Based on observation that many protein complexes typically have small diameter and average node distance, IPCA [23] modifies algorithm DPClus by considering subgraph diameter and interaction probability. The interaction probability of a node to a subgraph is defined as the number of edges between the node and subgraph normalized by the total number of nodes in the subgraph, and it is similar to cluster property and also closely related to subgraph density. The node weighing measure and seed selection strategy are identical to DPClus. In the sense of weighted networks, speed and performance in clustering (SPICi) [24] is proposed to handle the computation complexity of clustering large PPI networks. It builds clusters greedily, starting from local seeds that have high weighted degree, and greedily adding an adjacent unclustered node with the highest support score that maintains the density of the clusters. The cluster expansion approach of SPICi is simpler than DPClus and output is a set of disjoint dense subgraphs.

The study of protein complexes using affinity purification and mass spectrometry [25] suggests that major protein complexes contain a core in which proteins have relatively more interactions among themselves and each attachment protein binds to a subset of core proteins to form a complex. Based on this observation, ICSC [4] starts with a subgraph as a seed and then greedily adds nodes to find dense subgraphs. The definition of closeness of a node to a subgraph is the same as the interaction probability used in IPCA. Algorithms in this category include Core [26], COACH [10], GC-Coach [27] and WPNCA [28], while proteins are likely to have interactions with only one hub-protein within a few complexes that exhibit starlike structures in PPI networks [29,30].

PPI networks obtained from high-throughout biological experiments are noisy with false positive interactions. Taking into account the reliability of protein interactions, some efforts are made to identify protein complexes using the topology of PPI networks [31,32]. In order to generate robust clustering techniques, several computational approaches detect protein complexes from PPI networks integrating gene ontology (GO) annotation [33,34], genomic data [35] and so on.

Various graph clustering approaches have different clustering criteria to find local dense subgraphs and work well in detecting protein complexes from PPI networks. The local seed expansion method is among the most successful strategies for overlapping graph clustering [36]. However, there are still some limits in such algorithm: (1) measure the representability of a node to a cluster using only density of the subgraph induced by the node and its immediate neighborhood; (2) given a graph with weighted node, clusters are sensitive to the choice of the starting node [20]. Existing seeding strategies usually select a node with the highest weight as a starting node (seed) to find a cluster, without a process to adjust centers of clusters. This leads to a lack of diversity of algorithms; (3) existing closeness (interaction probability) of a node to a cluster only considers candidate nodes’ density or connections between the candidate nodes and the cluster.

In this article, we address the above limits and propose a new seed-expansion graph clustering algorithm (SEGC) that produces overlapped clusters for protein complex detection. It consists of three main phases: node weighing, seed selection and cluster expansion. In the stage of node weighing, SEGC combines different attribute information of node structure, and further improves the representability of nodes to a larger local neighborhood by an iterative weighing method. It has a diversity to adapt to different networks. In order to enhance the diversity of proposed algorithm, the roulette wheel is used to choose seed nodes of potential clusters. In the cluster expansion phase, a new closeness is proposed considering the influence of connections between a candidate node and a cluster on both the cluster and candidate node. We apply this clustering algorithm to cluster several PPI networks of *Saccharomyces cerevisiae*. The results show that SEGC outperforms other algorithms under full coverage in terms of both *F*-measure and accuracy with a real benchmark protein complex data set.

## 2. Preliminary

A protein-protein interaction (PPI) network can be represented by a graph *G* = (*V*, *E*) with node (protein) set *V* and edge set *E* that contains the edges (interactions) of the graph *G*. We consider only simple undirected graphs, which contain no self loops and multiple edges. Let *n* = *|V|* be the number of nodes and *m* = *|E|* be the number of edges. We denote an edge in *G* as an unordered pair ${(v}_{i}{,\;v}_{j})$ or $e_{\mathrm{ij}}$, where $v_{i}{,\;v}_{j}\;\in \;$*V*. A graph *H* = (*V*(*H*), *E*(*H*)) is called a subgraph of *G* if *V*(*H*) ⊆ *V* and *E* (*H*) ⊆ *E*, denoted as *H* ⊆ *G*. The diameter of a subgraph *H* is the largest length of a shortest path between any two nodes in subgraph *H*, written as *D*(*H*). An induced subgraph *G*[*S*] is a graph whose node set is *S* ⊆ *V* and whose edge set consists of all of the edges in *E* that have both endpoints in *S*. We write [*S*] to denote the induced subgraph by node subset *S* when without causing confusion. Table 1 lists the main symbols used in this paper.

Let *l* be a nonnegative integer. A path of length *l* from *u* to *v* in *G* is a sequence of *n* edges $e_{1}\;,\cdots {,\;e}_{l}$ of *G* for which there exists a sequence $x_{0\;}{=\;u,\;x}_{1}{,\;\ldots ,\;x}_{l-1}{,\;x}_{l}\;=\;v$ of mutually distinct nodes such that $e_{i\;}$ has, for *i* = 1, ..., *l*, the endpoints $x_{i-1}$ and $x_{i}$. We denote this path by its node sequence${\;x}_{0\;}{\ldots \;x}_{l}$. The distance of *u* and *v* is the length of the path between *u* and *v* in *G* such that the number of its edges is minimized.

The open neighborhood (or neighborhood) of a node *v*, denoted as $N_{G}(v)$ or *N*(*v*), is the subgraph induced by all nodes that are adjacent to *v*. The closed neighborhood is defined in the same way but also includes *v* itself, denoted as $N_{G}[v]$ or *N*[*v*]. Unless otherwise stated, we also use $N_{G}(v)$ (or) to represent the node set of $N_{G}(v)$ (or $\;N_{G}[v]$).

The 1-neighborhood of a given node $v_{i}\;\in \;V$ is represented by $\;N\left(v_{i}\right)\;=\;\left\{{\;v}_{j}\;\in {\;V\;|\;(v}_{i}{,\;v}_{j})\;\in \;E\right\}$, and then the set of *k*-neighborhood can be defined by

(1) $$N_{k}\left(v_{i}\right)\;=\;\{\begin{array}{ll} N\left(v_{i}\right), & \mathrm{if}\;k\;=\;1,\\ N_{k-1}\left(v_{i}\right)\;\cup \;\left\{v_{j}\;\in \;V\;|\;\mathrm{dis}\left(v_{i}{,\;v}_{j}\right)\;=\;k\;\right\},\; & \mathrm{if}\;k\;>\;1, \end{array}$$

where ${\mathrm{dis}\;(v}_{i}{,\;v}_{j})$ denotes the distance between $v_{i}\;$and $v_{j}$.

The degree *DC*(*v*) of a node *v* is the number of elements of $N_{G}(v)$, i.e., $\mathrm{DC}\left(v\right){=|\;N}_{G}(v)\;|$. The degree *DC*(*H*) of a node subset *H* is the sum of degree of the nodes of *H*, i.e.,$\;\mathrm{DC}\left(H\right)\;=\;\underset{v\in H}{\sum }\mathrm{DC}(v)$.

The goal of traditional graph clustering is grouping the nodes of a given input graph into *p* disjoint clusters (subgraphs) $C_{1}{,\;C}_{2},\;\cdots {,\;C}_{p}$ such that $\;V\left({\;C}_{1}\;\right)\;\cup \;V\left({\;C}_{2}\;\right)\;\cup \;\cdots \;\cup \;V\left({\;C}_{p}\right)\;=\;V\;$ and $\;V\left(C_{1}\right)\;\cap \;V\left(C_{2}\right)\;\cap \;\cdots \;\cap \;V\left({\;C}_{p}\right)\;=\;\emptyset$. For the problem of overlapping clustering in complex detection, the goal is to find clusters such that $\;V\left({\;C}_{1}\;\right)\;\cup \;V\left({\;C}_{2}\;\right)\;\cup \;\cdots \;\cup \;V\left({\;C}_{p}\right)\;\subseteq \;V$ and $\exists \;V\left(C_{i}\right)\;\cap \;V\left(C_{j}\right)\;\neq \;\emptyset \;$. A protein complex is usually abstracted as a connected subgraph in a PPI network and graph clustering is natural for protein complex detection. Here, graph clustering finds clusters within a given graph rather than the clustering between graphs.

## 3. Method

### 3.1. Algorithm Overview

We propose a new graph clustering algorithm based on seed-expansion approach (SEGC) to detect protein complexes using network topology attributes only. It consists of three main phases: node weighing, seed selection and cluster expansion. In the stage of node weighing, we compute the weights (i.e., representability) of nodes by a new metric. In seeding phase, the roulette wheel selection is used to find nodes with higher weight as seeds with probability proportional to their weights. In expansion phase, we expand the original seeds to form dense subgraphs as clusters based on a newly defined closeness measure (see Equation (8)). One could find a cluster by executing seed selection and cluster expansion. The seed of next cluster will be selected in nodes that have no cluster assignment. We do not remove any clustered node or edge to keep the original input graph complete. SEGC ensures that every node in PPI networks will be assigned into at least one predicted complex. SEGC can also obtain overlapping clustering, which means that some nodes might be attached to more than one cluster.

### 3.2. Node Weighing

In graph clustering, how to measure the representability of a node to a cluster by connections between nodes is a key issue. Let *w*(*v*) be the weight of a node v and be usually computed according to local information within a subgraph consisting of nodes *N*[*v*]. The node with higher *w*(*v*) has better representative to the subgraph *N*[*v*]. The most basic centrality measure is degree centrality (*DC*) based on the observation that the hub nodes usually have more edges [24,37]. There should be good clusters around high degree nodes in real-world networks with a power-law degree distribution. However, a node with a high degree is not enough to reflect the representability to a cluster [36,38]. In addition, the existing node importance metrics are mainly based on the structure information only within a node’s direct neighborhood. A good node weighing measure should reflect the importance of a node in a larger neighborhood of the node.

We proposed a new node weighing vector *W* to overcome the above shortcomings. It not only integrates topological attribute information of nodes and edges, but also gets importance of a node *v* within *k*-neighborhood of *v* (i.e., {*v*} ∪ $N_{k}(v)$) through *k* iterations, where *k* is a predefined parameter. A larger *k* indicates that the weight of node *v* represents the information of a larger neighborhood around it. Given attribute matrix $A\in {\;R}^{n\;\times \;q}$ of *n* nodes with *q* attributes and weight coefficient vector $\vec{\beta }\in R^{1\;\times \;q}$ of attributes, the node weight vector in *i*-th (1 ≤ i ≤ k) iteration is defined as

(2) $$W^{i}=A^{i-1}{\vec{\beta }}^{⊤},$$

where $A^{i-1}$ is the attribute matrix of nodes in (*i −* 1)th iteration, and element $w^{i}(v)$ of ${W\;}^{i},$ is determined as

(3) $$w^{i}(v)=A_{v}^{i-1}{\vec{\beta }}^{⊤}=\beta_{1}\cdot a_{1}^{i-1}(v)+\beta_{2}\cdot a_{2}^{i-1}(v)+\ldots +\beta_{q}\cdot a_{q}^{i-1}(v),$$

where $A_{v}^{i-1}{\;=\;[\;a}_{1}^{i-1}\left(v\right),\;\cdots {,\;a}_{q}^{i-1}(v)]$ and $\vec{\beta }=(\beta_{1},\ldots \beta_{q})$, which we will describe in detail in the following.

Let the weight of an edge *e* = (*u*, *v*) as the number of common neighbors between two ends of *e*, that is, τ(u, v) = |N(u) ∩ N(v)|. In order to reflect importance of a node *v* more comprehensive, we consider three basic attributes to calculate the weight of a node *v* in this paper, including: *DC*(*v*), the degree of node *v*; *DC*(*N*(*v*)), the degree of direct neighbors of *v*; and $\underset{u\in N(u)}{\sum }\tau (v,\;u)$, the sum of the weights of its incident edges. These three attributes can not only reflect the degree information of the node itself, but also the neighborhood information around the node. For convenience, we initialize the weight of a node as its degree, i.e., $w^{0}\left(v\right)\;=\;\mathrm{DC}(v)$. Therefore, we can define elements of the attribute vector of node *v* in *i*th (1 ≤ *i* ≤ *k*) iteration as:

(4) $$\begin{array}{ll} a_{1}^{i}\left(v\right) & =\;w^{i-1}\left(v\right),\\ a_{2}^{i}\left(v\right) & =\;\underset{u\in N\left(v\right)}{\sum }w^{i-1}\left(u\right)\\ a_{3}^{i}\left(v\right) & =\;\underset{u\in N\left(v\right)}{\sum }\tau \left(v,u\right). \end{array},$$

Since the significances of each attribute mentioned above are quite different from each other, we use weight coefficient vector $\vec{\beta \;}$ to weigh each attribute. The number of elements in $\vec{\beta \;}$ equals the number of attributes used in the calculation of node weights. Then, we have

(5) $$w^{i}\left(v\right){\;=\;\beta }_{1\;}\cdot {\;w}^{i-1}\left(v\right){\;+\;\beta }_{2}\;\cdot \;\underset{u\in N(v)}{\sum }w^{i-1}{(u)+\;\beta }_{3}\;\cdot \;\underset{u\in N(v)}{\sum }\tau (v,\;u),$$

where $\beta_{1\;}{+\;\beta }_{2\;}{+\;\beta }_{3\;}=\;1$.

The first item of Equation (5) denotes the centrality information in *i*th iteration of the node itself. The second item reflects the centrality information of its adjacent nodes in *i*th iteration. The third item adds weights of its incident edges to the centrality information of node *v*. If the weights of its incident edges are relatively high, then the node *v* might be a meaningful point for local module searches in functional networks, similar to [24].

From the definition of the node weight, it can also be obtained that the nodes with higher weight should be more representative for its local topological neighborhood. The number of iteration determines the range that the node weight can reflect. For example, in the first iteration, the node weight reflects the direct neighborhood including its adjacent nodes and its incident edges; however, in the *i*th iteration, the node weight can reflect the *i*-neighborhood of node *v.* If *i* is the diameter of a graph *G*, then $w^{i}(v)$ can measure the centrality of node *v* in the range of the whole network.

Since $\beta_{1}{\;+\;\beta }_{2}{\;+\;\beta }_{3}\;=\;1$, the node weight defined in Equation (5) can also be formulated as:

(6) $$w^{i}\left(v\right){\;=\;\beta }_{1}\cdot {\;w}^{i-1}\left(v\right){\;+\;\beta }_{2}\cdot \;\underset{u\in N\left(v\right)}{\sum }w^{i-1}\left(u\right)\;+\;\left(1\;-{\;\beta }_{1\;}-{\;\beta }_{2}\right)\cdot \;\underset{u\in N\left(v\right)}{\sum }\tau \left(v,\;u\right).$$

If $\beta_{1}=\;1$, we have $w^{i}(v)\;=\;\mathrm{DC}(v)$ and the representative of a node is determined only by its degree. If $\beta_{2}\;=\;1$, the representative of a node is determined by the degree of the *i*th neighborhood of node *v*. If ${\;\beta }_{3}\;=\;1$, weights of direct edges of a node is a key to measure local importance, in this case, the node weight $w^{i}(v)\;=\;\underset{u\;\in \;N(v)}{\sum }\tau (v,\;u)$ which is the same as that defined by DPClus [22].

**Figure 1:**
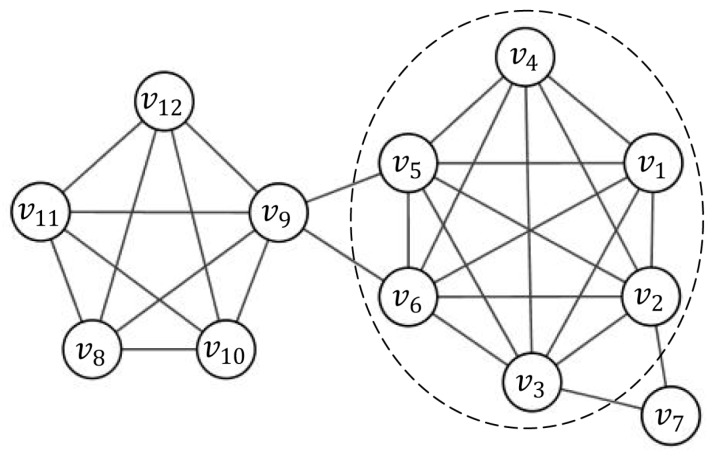
An example network. Although node $v_{2}$, $v_{5}$ and $v_{9}$ have the same degree, they have different representability to a subgraph from Equation (5).

The linear combination of the three parts above makes the representability of nodes to a subgraph more complete. As shown in Figure 1, both node $v_{2}$ and node $v_{5}$ lie in the complete subgraph induced by ${\{v}_{i}|1\;\leq \;i\;\leq \;6\}$ and have the same degree, i.e., $w^{0}{(v}_{2}{)\;=\;w}^{0}{(v}_{5})\;=\;6$. However, node $v_{5}$ lies in a more important position than $v_{2}$ since the 2-neighborhood of $v_{5}$ includes some nodes of the dense subgraph induced by $\left\{v_{i}|8\;\leq \;i\;\leq \;12\right\}$. From Equation (5) with weight coefficient vector (0.2, 0.6, 0.2), we have $w^{1}{(v}_{2})\;=\;23.6$, $w^{1}{(v}_{5})\;=\;26$ and $w^{2}{(v}_{2})\;=\;86.2$, $w^{2}{(v}_{5})\;=\;92.9$. Therefore, $v_{5}$ has better representative than $v_{2}$ when $\beta_{1}\;=\;0.2,\beta_{2}\;=\;0.6,\beta_{3}\;=\;0.2$.

### 3.3. Seed Selection

The seed of a cluster should have a better representative for the cluster, which indicates that the weight of the seed node should be relatively larger than other nodes in the cluster. However, the node with the largest weight might not always be the best choice for the seed of the considered cluster. In order to improve the diversity of seed selection, SEGC uses a roulette wheel to select seeds from the perspective of probability. The probability of a node v ∈ V as a seed is defined as:

(7) $$\;P(v)\;=\;\frac{{{[w}^{i}(v)]}^{2}}{\sum_{x\in V}{{[w}^{i}(x)]}^{2}}.$$

The larger the weight is, the larger the probability that the node will be selected as a seed.

At the beginning, our algorithm picks some node *v* as a seed and extends it to a cluster *C*(*v*) using the cluster expansion process described in next section. Once the cluster *C*(*v*) is obtained, we begin to select the seed node for next cluster. The seed node of the next cluster should be away from the existing seeds in order to reduce generation of redundant clusters. Hence, all nodes in existing clusters are no longer selected as seed nodes. However, every node might be a member of other clusters to form overlapping clusters. Thus, we choose seed nodes in the unclustered nodes that have not been included in any of predicted clusters by roulette wheel. The entire procedure of the approach terminates when there are no unclustered nodes.

### 3.4. Cluster Expansion

After obtaining a seed node *v*, we extend it to a cluster *C*(*v*), which initially consists of only the node *v*. The candidate node set for current *C*(*v*) is *N*(*C*(*v*)), the neighbors of *C*(*v*). For a candidate node *u*, we use the adjacent nodes of *u* in *C*(*v*) to determine the priority of whether *u* can be extended to *C*(*v*). We take into account both the proportion of N(u) ∩ C(v) in the node set of *C*(*v*) and the proportion of N(u) ∩ C(v) in the neighborhood of *u*. The priority of a candidate node u to cluster *C*(*v*) is defined as follows:

(8) $$\;\mathrm{NC}\left(u,\;C\left(v\right)\right)\;=\;\lambda \;\frac{\left|N\left(u\right)\;\cap \;V\left(C\left(v\right)\right)\right|}{\left|V\left(C\left(v\right)\right)\right|}\;+\;(\;1\;-\lambda )\;\frac{\left|N\left(u\right)\;\cap \;V\left(C\left(v\right)\right)\right|}{\left|N\left(u\right)\right|}.$$

The NC(u, C(v)) measures how strongly a node *u* is connected to a cluster *C*(*v*). For a dense cluster, a node connects to most of the nodes in the cluster. For the nodes lying on the spare periphery of a cluster, most of their neighbors are in the cluster. The first item of Equation (8) represents the effect of the size of current cluster *C*(*v*), *V(C*(*v*)) is the node set of the subgraph *C*(*v*) with node *v* as the seed node. The second item represents the effect of the size of the neighborhood of *u*. The priority of a candidate node *u* to a cluster *C*(*v*) is positively correlated to the number of adjacent nodes of *u* in *C*(*v*), negatively correlated to the number of nodes in *C*(*v*), and negatively correlated to the number of neighborhood of *u*.

The parameter λ ∈ [0, 1] in Equation (8) is to control the priority of *u* to C(*v*) during the expansion process. When λ > 0.5, the first item of Equation (8) plays a determining role for NC (u, C(v)). We might obtain a relatively dense cluster with a larger λ, since we give preference to nodes with more connections with the current cluster. In addition, we might obtain a sparse cluster with a smaller λ since we give preference to nodes with a low degree. A cluster should be denser around its seed and might be not so dense away from the seed, so we should set a larger λ in the beginning of the expansion. With the increase of the number of nodes in the cluster, we should set a smaller λ to allow nodes lying on the periphery of cluster could be found. Hence, we set $\lambda \;=\;\frac{1}{r\;\times \;\sqrt{V(C(v))-1\;}+\;1}$, where r is a predefined parameter to control the reducing rate of λ.

Considering the network shown in Figure 1 as an example, let ${C(v}_{5})$ be the induced subgraph by node set ${\{v}_{i}|1\;\leq \;i\;\leq \;6\}$ and the seed node is $v_{5}$. The candidate node $v_{7}$ should be a periphery node of ${C(v}_{5})$ and should be included in the current cluster. Another candidate node $v_{9}$ might not be a member of ${C(v}_{5})$. If the threshold of priority is set to 0.5, candidate node $v_{7}$ is added to ${C(v}_{5})$, whereas candidate node $v_{9}$ will not be added.

Based on the study of known complexes in protein networks, most complexes have a very small subgraph diameter [23,39]. Thus, we have two parameters ε and θ for node priority and graph diameter, respectively. That is to say, for a candidate node *u* and a cluster *C*(*v*), if NC(u, C(v)) > and D([C(v) ∪ {u}]) ≤ θ, node *u* would be added into cluster *C*(*v*), and then C(v) = C(v) ∪ {u}. The expansion progress would end when we could not find node *u* in *N*(*C*(*v*)) satisfying NC(u, C(v)) > and D([C(v) ∪ {u}]) ≤ θ.

### 3.5. Complexity

We repeat the seed selection and cluster expansion process until all nodes in a graph are clustered. The frame of the proposed approach SEGC are given in Algorithm 1. Let *G* = (*V*, *E*) be the graph corresponding to the considered protein interaction network with node set *V* and edge set *E*, |*V*| = *n* and |*E*| = *m*. Then, the average computational cost for computing edge weights is $O\;(\bar{d}\;\times \;m)$, where $\bar{d}$ is the average degree of *G*. It takes $O\;(k\;\times \bar{\;d}\;\times \;n)\;=\;O\;(k\;\times \;m)$ time to obtain node weights for *k* iterations.

It needs O (n) time to select one seed, and O (|C| × n) to select all seeds for |*C*| clusters. The algorithm obtains a cluster *C* from its seed. During the expansion process of *C*, it should take O(|V(C) ∪ N(V(C))| × log[|V(C) ∪ N(V(C))|]) time to compute *NC*(*x*, *C*) for each node x ∈V(C) ∪ N(V(C)) and sort them in nondecreasing order. For the worst case, *C* might include all nodes of the considered network, that is to say, we need O( n × log n) time to obtain a cluster and need O(|C| × n × log n) time in total for cluster expansion. Thus, the time consumed for algorithm SEGC is O(|C| × n × log n).

**Algorithm 1.** A seed-expansion graph clustering method (SEGC).**Input:** A given graph *G* = (*V*, *E*), parameters $\beta_{1}{,\;\beta }_{2},\;k,\;r,\;\varepsilon$ and θ.**Output:** A set of clusters${\;S\;=\;\{C}_{1},\;\cdots {,\;C}_{p}\}$.1: S = ∅;2: For each node v ∈ V, let $w^{0}(v)\;=\;\mathrm{DC}(v)$; //*DC*(*v*) is degree centrality of node *v*.3: For each edge (v, u) ∈ E, let τ(v, u) = |N(v) ∩ N(u)|;4: **for**
*i* = 1 to *k*
**do** //(***Node Weighing***)5: For each node  v ∈ V let$w^{i}(v)\;=\;\beta_{1}\;\cdot {\;w}^{i-1}(v)\;+\;\beta_{2}\;\cdot \underset{u\in N(v)}{\sum }w^{i-1}(u)\;+\;(1\;-{\;\beta }_{1}\;-\beta_{2})\;\cdot \underset{u\in N(v)}{\sum }\tau (v,\;u);$6: **end for**7: For each node v ∈ V, compute the selection probability $\;P\left(v\right)\;=\;\frac{{{[w}^{i}(v)]}^{2}}{\sum_{x\in V}{{[w}^{i}(x)]}^{2}}\;;$8: **while**
$|V\;-\;U_{C\in S}V(C)\;|\;>$
**do**9: Select a seed node *v* using roulette wheel; //(***Seed Selection***)11: *C*(*v*) = **Cluster Expansion**({*v*}); //(***Cluster Expansion***)12: S = S ∪ C(v);13: **end while** **Subroutine Cluster Expansion(***C***)**1: Let N′(C) = {x|x ∈ N(C), NC(u, C) > D([C ∪ {u}]) ≤ θ};2: **if**
N′(C) ≠ ∅
**then**3: Let $u=a\underset{x\;\in \;N\prime (C)}{\text{rg max}}\{\mathrm{NC}(x,\;C)\}$ be the node with highest priority;4: C = C ∪ {u}; 5: *C* = **Cluster Expansion**(*C*) 6: **else** 7: **Return**
*C* 8: **end if**

## 4. Experiments and Results

We implemented the proposed SEGC algorithm in C++ on Microsoft Visual Studio 2010 (Redmond, WA, USA). SEGC has been successfully executed and tested on Windows 7 platform (Microsoft Corporation, Redmond, WA, USA), running on a PC with Intel Core CPU (Santa Clara, CA, USA) i7-2600@3.40 GHz and 8 GB RAM.

### 4.1. PPI Datasets and Metrics

We use *Saccharomyces cerevisiae* as an experimental organism, which is one of the most popular species, because it is one of the earliest research objects and has the most abundant PPI data. Five PPI networks of *Saccharomyces cerevisiae* are used and marked as Gavin02 [6], Gavin06 [25], Krogan_core, Krogan_extend [40] and BioGrid, respectively. These data sets are widely used in protein complex detection. Gavin02 includes 1352 proteins and 3210 interactions. Both Gavin06 and Krogan_extend are tandem affinity purification (TAP) data that include 1430 proteins with 6531 interactions and 3672 proteins with 14,317 interactions, respectively. Krogan_core contains only highly reliable interactions among Krogan_extend. BioGrid is constructed by all of low-throughput physical interactions in BioGRID database [41] (version 3.4.137) and includes 4254 proteins and 21,375 interactions. Table 2 shows the information of the five networks above. The density of a graph *G* = (*V*, *E*) is the ratio of the total number of edges to the total number of all possible links between all nodes, and is defined as *Density*(*G*) = 2|*E*|/(|*V*|(|*V*|−1)). We consider only a simple graph in this paper, so we remove all self-interactions and duplicate interactions.

We take CYC2008 [42] as gold standard complex set to evaluate protein complexes predicted by the proposed algorithm SEGC. There are 408 manually curated complexes in CYC2008. Each protein complex in CYC2008 is reported by small-scale experiments and is of high reliability, so CYC2008 has been used as a benchmark set by many computational approaches for the prediction of protein complexes.

To assess the quality of results obtained by different algorithms, we use several evaluation criteria including precision, recall, *F*-measure, clustering-wise positive predictive value (PPV), clustering-wise sensitivity (Sn) and accuracy.

*F*-measure is the most widely used metric [28,43,44], and can evaluate both the accuracy of clusters matching known protein complexes and the accuracy of the known complexes matching the predicted clusters. Given a predicted cluster set ${C\;=\;\{C}_{1}{,\;C}_{2}{,\;\ldots ,\;C}_{p}\}$ and the gold standard complex set ${\mathrm{CO}\;=\;\{\mathrm{CO}}_{1}{,\;\mathrm{CO}}_{2}{,\;\ldots ,\;\mathrm{CO}}_{q}\}$, the neighborhood affinity score ${\mathrm{NA}(C}_{i}{,\;\mathrm{CO}}_{j})$ between a predicted cluster $C_{i}$ and a standard complex ${\mathrm{CO}}_{j}$ in benchmark set is defined as

(9) $${\mathrm{NA}(C}_{i}{,\;\mathrm{CO}}_{j})\;=\;\frac{{|C}_{i}\cap {\mathrm{CO}}_{j}{|}^{2}}{{|C}_{i}{|\;\times \;|\mathrm{CO}}_{j}|},$$

for i ∈{1, 2, …, p} and j ∈{1, 2, …, q}.

The neighborhood affinity score ${\mathrm{NA}(C}_{i},\;{\mathrm{CO}}_{j})$ quantizes the closeness between two complexes $C_{i}$ and ${\mathrm{CO}}_{j}$. The larger the $\mathrm{NA}(C_{i}{,\;\mathrm{CO}}_{j})$ is, the closer $C_{i}$ and ${\mathrm{CO}}_{j}$ are. If ${\mathrm{NA}(C}_{i}{,\;\mathrm{CO}}_{j})\;\geq \;\mu$, then $C_{i}$ and ${\mathrm{CO}}_{j}$ are considered to be matching, where μ is predefined threshold and is usually set to 0.2 [27,43]. We also set μ = 0.2 in this paper.

Let $M_{C}$ be the predicted cluster set such that every item in it matches at least one standard complex in CO, i.e.,

(10) $$M_{C}{\;=\;\{C}_{i}{|C}_{i}\in C\;\wedge \;\exists {j(\mathrm{CO}}_{j}\in \mathrm{CO}\;\wedge {\;\mathrm{NA}(C}_{i}{,\;\mathrm{CO}}_{j})\;\geq \;\mu )\}.$$

Let $M_{\mathrm{CO}}$ be the standard cluster set such that every item in it matches at least one predicted complex in C, i.e.,

(11) $$M_{\mathrm{CO}}{\;=\;\{\mathrm{CO}}_{j}{|\mathrm{CO}}_{j}\;\in \;\mathrm{CO}\;\wedge \;\exists {\;i(C}_{i}\;\in \;C\;\wedge {\;\mathrm{NA}(C}_{i}{,\;\mathrm{CO}}_{j})\;\geq \;\mu )\}.$$

The **precision** and **recall** are defined as follows:

(12) $$\mathrm{Precision}\;=\;\frac{{|M}_{C}|}{\left|C\right|},$$

(13) $$\mathrm{Recall}\;=\;\frac{{|M}_{\mathrm{CO}}|}{\left|\mathrm{CO}\right|}.$$

***F*-measure** is the harmonic mean of precision and recall to quantize the closeness between predicted complex set and standard complex set:

(14) $$F-\mathrm{measure}\;=\;\frac{2\;\times \;\mathrm{Precision}\;\times \;\mathrm{Recall}}{\mathrm{Precision}\;+\;\mathrm{Recall}}.$$

Let T be a p × q matrix, where row i corresponds to a cluster $C_{i}$ and column j corresponds to an annotated complex ${\mathrm{CO}}_{j}$. In addition, the element $T_{\mathrm{ij}}$ of T is the number of proteins that are in common between $C_{i}$ and ${\mathrm{CO}}_{j}$, i.e., $T_{\mathrm{ij}}{\;=\;|C}_{i}\;\cap {\;\mathrm{CO}}_{j}|$. The clustering-wise positive predictive value (*PPV*) is defined as:

(15) $$\mathrm{PPV}\;=\;\frac{\sum_{i\;=\;1}^{p}\sum_{j\;=\;1}^{q}\left(T_{\mathrm{ij}}\;\times \;\underset{j\;=\;1}{\overset{q}{\max}}{(T}_{\mathrm{ij}}/\sum_{j\;=\;1}^{q}T_{\mathrm{ij}})\right)}{\sum_{i\;=\;1}^{p}\sum_{j\;=\;1}^{q}T_{\mathrm{ij}}}.$$

The **clustering-wise sensitivity** (*Sn*) is defined as:

(16) $$\;\mathrm{Sn}\;=\;\frac{\sum_{j\;=\;1}^{q}\left({|\mathrm{CO}}_{j}|\;\times \underset{i\;=\;1}{\overset{p}{\;\max}}{(T}_{\mathrm{ij}}{/|\mathrm{CO}}_{j}|)\right)}{\sum_{j\;=\;1}^{q}{|\mathrm{CO}}_{j}|},$$

where ${|\mathrm{CO}}_{j}|$ is the number of proteins in complex ${\mathrm{CO}}_{j}$.

Accuracy is another important criteria to evaluate the accuracy of a prediction [33,45]. It can be obtained by the geometrical mean of the *PPV* and the *Sn* as follows:

(17) $$\mathrm{Accuracy}\;=\;\sqrt{\;\mathrm{PPV}\;\times \;\mathrm{Sn}}.$$

It is important for a clustering technique to cover all the nodes of a PPI network as clusters can be both dense and sparse. This will ensure that important functional modules or protein complexes are not missed during the clustering process [16]. The **Coverage** of an algorithm can be calculated as

(18) $$\;\mathrm{Coverage}\;=\;\frac{\left|\cup_{i\;=\;1}^{p}{V(C}_{i})\right|}{n}.$$

### 4.2. Parameter Setting

The proposed algorithm SEGC has six predefined parameters, weight coefficients $\beta_{1}$ and $\beta_{2}$ of node attributes, the number of iterations k, reduce rate r, closeness threshold ε and diameter threshold θ.

Weight coefficients $\beta_{1}$ and $\beta_{2}$ are used to compute the weights of nodes through k iterations. The parameters r, ε and θ are used in the cluster expansion process. We could find small dense clusters with less periphery nodes with smaller r or larger ε. Diameter threshold θ is to control the diameter of the found clusters.

**Figure 2:**
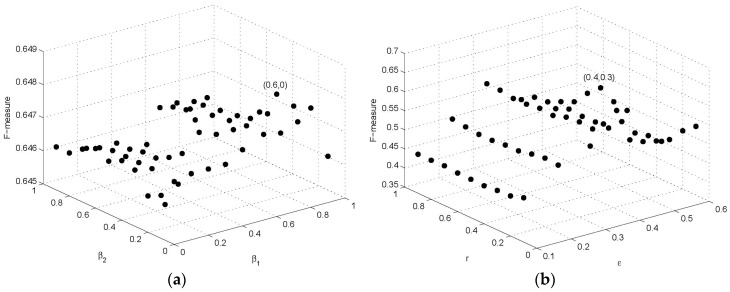
The effect of parameters on the performance of seed-expansion graph clustering (SEGC) on BioGrid: (**a**) the effect of $\beta_{1}$ and $\beta_{2}$; (**b**) the effect of r and ε.

BioGrid is a standard protein interaction network data set, in which all interactions are constructed by all of low-throughput physical interactions with high reliability and precision. Thus, we apply alternating direction method on BioGrid to obtain suggested values of these parameters, using *F*-measure as an optimization goal. We first fix $\beta_{1}$ = 0, $\beta_{2}$ = 0, *k* = 1, and the experiments on BioGrid PPI network with ε from 0.1 to 0.9, *r* from 0.1 to 0.9 were carried out to verify the influence of parameters ε and *r*. The *F*-measure reaches its maximum value when ε is 0.4 and r is 0.3. Then, we fix ε = 0.4, *r* = 0.3, and the *F*-measure is maximized at $\beta_{1}$ = 0.6, $\beta_{2}$ = 0, and *k* = 3. Next, we fix $\beta_{1}$ = 0.6, $\beta_{2}$ = 0, *k* = 3 and, in turn, try different values of parameters ε and *r*, and the experiments also obtain the best performance at ε = 0.4, *r* = 0.3. Therefore, in this study, we set $\beta_{1}$ = 0.6, $\beta_{2}$ = 0, *k* = 3, ε = 0.4, *r* = 0.3. Figure 2a shows the results of parameters $\beta_{1}$ and $\beta_{2}$ on *F*-measure with *r* = 0.3, ε = 0.4, and the effect of parameters r and ε on *F*-measure is shown in Figure 2b with $\beta_{1}\;=\;0.6$, $\beta_{2}\;=\;0$ and k = 3. We set diameter threshold θ = 2 since diameters of most known complexes are relatively small [35]. Thus, we finally decide to set the parameters ($\beta_{1}$, $\beta_{2}$, *k*, ε, *r*, θ) of SEGC to default values (0.6, 0, 3, 0.4, 0.3, 2), respectively, in all following experiments unless otherwise noted. 

### 4.3. Effectiveness of Our Strategies

We use algorithm IPCA [23] as the basic frame to test the effectiveness of each our strategies, such as node weighing in Section 3.2, roulette wheel in seed selection in Section 3.3 and priority definition in Equation (8) in Section 3.4.

We replace the definition of node weights in IPCA with Equation (5) proposed in Section 3.2, and the parameters in Equation (5) are set to $\beta_{1}\;=\;0.6$, $\beta_{2}\;=\;0$ and k = 3. For convenience, we name the IPCA algorithm with new node weighing method as IPCA-node weighing (NW). We add the roulette wheel method to seed selection in IPCA (named as IPCA-RW) and the results are shown in 5th column in Table 3. Because of the stochastic nature of the selection step, we run the procedure 500 times and choose the best clustering solution in usual practice. We replace the interaction probability (IN) in IPCA with the priority definition according to Equation (8) to obtain algorithm IPCA-NC, where r = 0.3, ε = 0.4 and θ = 2.

Table 3 shows the comparison results with original IPCA. It can be seen that each strategy can improve the performance of IPCA to a certain extent.

### 4.4. Comparison with Other Algorithms

We compare SEGC with other overlapping protein complexes detection methods: CFinder [18], DPClus [22], IPCA [23], Core [26], soft regularized Markov clustering (SR-MCL) [44], PE-measure and weighted clustering coefficient (PEWCC) [31], detecting complex based on uncertain graph model (DCU) [32], weighted COACH (WCOACH) [34] and weighted edge based clustering (WEC) [35]. Table 4 exhibits parameters of each algorithm, which are recommended by authors. Table 5 shows comparison results of all algorithms on five PPI networks: Gavin02 [6], Gavin06 [25], Krogan_core, Krogan_extend [40] and BioGrid.

Algorithms CFinder, SR-MCL and WEC produce less clusters that are so dense that the number of edges in clusters are nearly the same as that in complete subgraphs, so they have comparatively higher precision than other algorithms. A shorting coming of CFinder, SR-MCL and WEC is the loss of coverage especially on sparse networks. A small coverage usually yields small recall.

DPClus adopts a seed expansion strategy to find clusters, where the density of the cluster determines whether a node be included into the current cluster. Thus, DPClus could find many small dense clusters. The average number of nodes in a predicted cluster of DPClus is the smallest among all 10 experimental algorithms and is usually not bigger than five. This leads to the highest PPV among all algorithms and a higher coverage on the sparse network than CFinder and SR-MCL. Since DPClus removes the nodes and related edges from the considered network after obtaining a cluster, there might be some isolated nodes in the remaining network. Hence, DPClus could not obtain full coverage results. Core extends a cluster from several core proteins. If a candidate node connects with at least half of the nodes in a considered cluster, it would be added into the cluster. Hence, the size of clusters found by Core is usually larger than those found by DPClus, and the density of the found clusters is lower than DPClus. Therefore, Core always has a higher coverage than DPClus.

IPCA adopts also a seed expansion strategy as DPClus. IPCA keeps all nodes and edges in the network during the cluster extension process, and can obtain full coverage results. Our SEGC tries to find a better seed by using the roulette wheel strategy. It also considers both the density of the cluster and the connections between candidate nodes and considered the cluster in cluster extension process. Hence, SEGC improves the efficient of IPCA and can also obtain full coverage. It is also clear that SEGC performs better than the other nine methods in terms of *F*-measure and accuracy. The *F*-measure of SEGC is the highest on Gavin02, Krogan_core, Krogan_extend and BioGrid, and the accuracy of SEGC on Gavin02, Krogan_core and Krogan_extend is also the highest.

DCU and WCOACH produce huge clusters with a good coverage. Since a good fraction of each complex is covered by these huge clusters, DCU and WCOACH have a high Sn. The clusters generated by PEWCC are usually smaller than the ones produced by DCU and WCOACH; thus, PEWCC has a better PPV. 

It is worth noting that our SEGC has a poor performance on Gavin06. It is because that we use default parameters on Gavin06 such as $\beta_{1}\;=\;0.6$, $\beta_{2}\;=\;0$, k = 3, r = 0.3, ε = 0.4 and θ = 2. The parameter ε is to control the density of considered clusters. We adopt ε = 0.4 as default by executing experiments on the BioGrid dataset. The density of the network from BioGrid is 0.0023, which is likely as those from Gavin02 (0.0035), Krogan_core (0.0019) and Krogan_extend (0.0021) datasets. However, the density of network obtained from Gavin06 is 0.0064, which is almost triple the density of others. This means that the nodes in the Gavin06 PPI network have more connections between them, and the protein complexes existing in Gavin06 PPI network may be denser than clusters obtained from the other PPI networks. A lower value of ε cannot accurately measure the closeness within clusters from Gavin06. For ε = 0.4, SEGC obtains predicted clusters with much more nodes in them and has a not so good performance in Gavin06. If we improve ε to 0.55, SEGC would get denser clusters and obtain a better performance, as shown in Table 5 (values in parentheses).

### 4.5. Stability of SEGC

For seed selection, SEGC repeats the selection procedure a few times with a probabilistic approach, the roulette wheel. The average clustering performance with variances on each data set is summarized in Table 6. We find SEGC always gives a very small variance for each criteria. It means that our algorithm has a good stability. The stability of SEGC is based on two reasons. First, the seed selection process is not completely random. Second, there is a positive correlation between the weight w(v) of a node *v* and the probability P(v) that the node will be selected as a seed. Equation (7) further improves the positive correlation by increasing the node weight w(v) to ${\left[w(v)\right]}^{2}$. Compared to w(v), ${\left[w(v)\right]}^{2}$ increases the inhomogeneity of probability *P*, and the ordering of the probabilities is not disturbed.

### 4.6. Examples of Predicted Complexes

**Figure 3:**
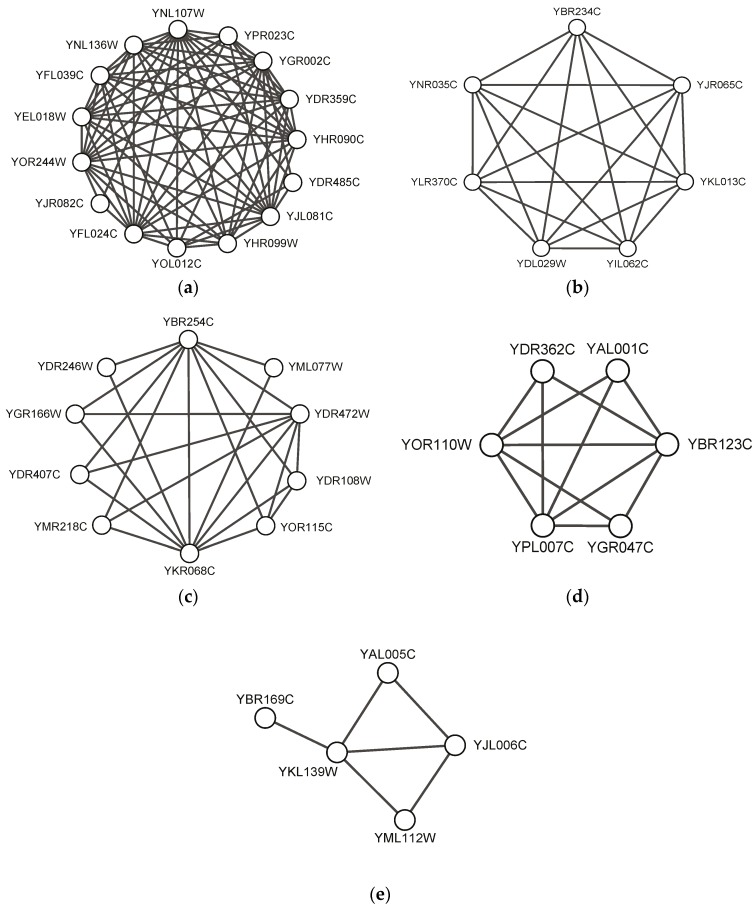
Examples of predicted complexes matching standard complexes: (**a**) NuA4 histone acetyltransferase complex predicted by SEGC on BioGrid; (**b**) Arp2/3 protein complex predicted by SEGC on Gavin02; (**c**) transport protein particle (TRAPP) complex predicted by SEGC on Gavin06; (**d**) transcription factor TFIIIC complex predicted by SEGC on Krogan_extend; (**e**) carboxy-terminal domain protein kinase complex predicted by SEGC on Gavin06.

We exhibit some predicted protein complexes obtained by our SEGC in this section. Figure 3 visualizes five predicted complexes, which completely match standard complexes in CYC2008. There are 100 predicted protein complexes found by our SEGC, which completely match standard complexes in CYC2008 in total. Figure 3 shows five typical protein complex examples such as NuA4, Arp2/3, TRAPP, Transcription factor TFIIIC and Carboxy-terminal domain protein kinase. It can be seen that the proposed algorithm SEGC could find both dense complexes close to the complete subgraph (see Figure 3a–c) and sparse complexes (see Figure 3d–e). In particular, SEGC could find complexes with pendant nodes whose degree is 1 in protein networks, as shown in Figure 3e.

**Figure 4:**
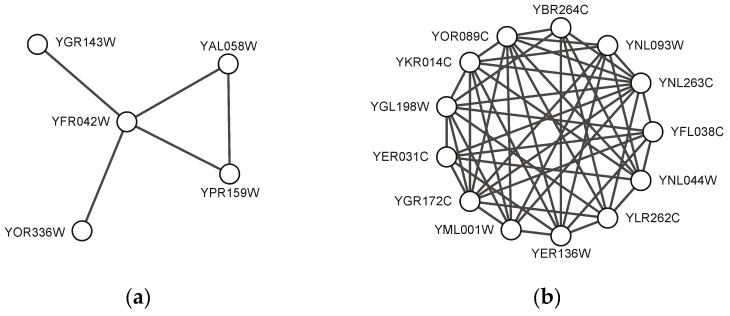
Examples of predicted complexes in which none of proteins is labeled by any of standard complexes: (**a**) a predicted complex by SEGC on BioGrid; (**b**) another predicted complex by SEGC on BioGrid.

Figure 4 shows two predicted complexes with similar topological structure as found protein complexes, which indicates that they might be potential protein complexes. These might give some useful information for detecting new protein complexes in the future. Table 7 shows gene ontology annotation information and the corresponding *p*-value of the examples shown in Figure 3 and Figure 4.

## 5. Conclusions

Graph clustering has significant popularity in bioinformatics as well as data mining research, and is an effective approach for protein complex identification in protein interaction networks. In this article, we proposed a seed expansion graph clustering algorithm SEGC for protein complex detection in protein interaction networks. SEGC weights nodes by multi-attribute fusion, selects seed nodes using the roulette wheel, and extends a cluster by considering both the density of the cluster and the connection of candidate node itself. It gets a soft clustering under full coverage of the entire network. Compared with other protein complex detection algorithms, SEGC shows a comparable performance in terms of precision, recall, *F*-measure, clustering-wise positive predictive value (PPV), clustering-wise sensitivity (Sn) and accuracy. 

There are still some problems that need further study. In large PPI networks, it is imperative for clustering techniques to find important nodes (e.g., seed nodes) more accurately, while the computation complexity of clustering algorithms can be handled. In addition, suitable non-topological information will help to reduce the noise of data. The combination of non-topological and topological attributes might improve the performance of clustering algorithms.

## Figures and Tables

**Table 1:** Description of the main symbols used in this paper.

Symbol	Description
*G* = (*V, E*)	A graph G including a node set *V* and an edge set *E*
*n*	The number of nodes in a graph
*m*	The number of edges in a graph
$v_{i}$	The *i*th node in *V*
${(v}_{i}{,\;v}_{j})$ or $e_{\mathrm{ij}}$	The edge in *E* between node $v_{i}\;$ and $v_{j}$
${\mathrm{dis}\;(v}_{i}{,\;v}_{j})$	The distance between node $v_{i}$ and $v_{j}$
$N_{k}$	*k*-neighborhood
*V*(*S*)	The node set of a subgraph *S*
*A*	The attribute (feature) matrix of nodes in a graph
	The weight vector of the node attributes
*k*	The maximum number of iterations
*W*	The weight matrix of nodes
*w*(*.*)	The weight of a node or an edge
*P*(*v*)	Probability of node *v* being selected
*C*(*v*)	The cluster (subgraph) with node *v* as the seed
*NC*(*u*, *S*)	The closeness between node u and subgraph *S*
λ	The parameter to control two items in *NC*
r	Reduce rate of λ
*D*	Diameter of a graph
ε	The user-defined threshold of *NC*
θ	The user-defined threshold of diameter

**Table 2:** Protein-protein interaction (PPI) datasets.

Items	Gavin02	Gavin06	Krogan_Core	Krogan_Extend	BioGrid
Proteins	1352	1430	2708	3672	4187
Interactions	3210	6531	7123	14317	20454
Density	0.0035	0.0064	0.0019	0.0021	0.0023
Throughput	High	High	High	High	Low

**Table 3:** Comparison results of IPCA algorithm with new node weighing method (IPCA-NW), IPCA algorithm with roulette wheel method (IPCA-RW) and (IPCA algorithm with *NC* metric (IPCA-NC) in Equation (8)) with original IPCA.

Network	Criteria	IPCA	IPCA-NW	IPCA-RW	IPCA-NC
Gavin02	Precision	0.4675	0.4686	0.4851	0.5462
	Recall	0.3505	0.3505	0.3505	0.3603
	*F*-measure	0.4006	0.4010	0.4070	0.4342
	PPV	0.5541	0.5532	0.5522	0.5578
	Sn	0.3646	0.3646	0.3646	0.4141
	Accuracy	0.4495	0.4491	0.4487	0.4806
Gavin06	Precision	0.5289	0.5298	0.5460	0.4603
	Recall	0.3750	0.3750	0.3750	0.3750
	*F*-measure	0.4389	0.4392	0.4446	0.4133
	PPV	0.5375	0.5375	0.5447	0.5299
	Sn	0.4807	0.4807	0.4797	0.5021
	Accuracy	0.5083	0.5083	0.5112	0.5158
Krogan_core	Precision	0.4732	0.4744	0.4857	0.4769
	Recall	0.5662	0.5637	0.5686	0.5735
	*F*-measure	0.5155	0.5152	0.5239	0.5208
	PPV	0.6058	0.6054	0.6037	0.6164
	Sn	0.5786	0.5776	0.5792	0.5891
	Accuracy	0.5921	0.5913	0.5913	0.6026
Krogan_extend	Precision	0.4114	0.4120	0.4185	0.4434
	Recall	0.4926	0.4926	0.4951	0.5466
	*F*-measure	0.4484	0.4487	0.4536	0.4896
	PPV	0.5234	0.5250	0.5304	0.5499
	Sn	0.5974	0.5974	0.5979	0.6135
	Accuracy	0.5592	0.5600	0.5631	0.5809
BioGrid	Precision	0.5075	0.5083	0.5135	0.5316
	Recall	0.8088	0.8088	0.8088	0.8260
	*F*-measure	0.6237	0.6243	0.6282	0.6469
	PPV	0.4482	0.4480	0.4485	0.4748
	Sn	0.7885	0.7885	0.7880	0.8115
	Accuracy	0.5945	0.5944	0.5945	0.6207

**Table 4:** Parameters of each algorithms.

Algorithm	Parameter	Value
CFinder	*k*-clique template	3
DPClus	cluster property value	0.5
	density	0.7
IPCA	interaction probability	0.4
	diameter	2
SR-MCL	inflation	2
	balance	0.5
	iterations	30
	penalty ratio	1.25
	quality function	1.2
	overlap threshold	0.6
PEWCC	join parameter	0.5
	overlap threshold	0.8
DCU	expected density	0.2
WCOACH	neighborhood affinity threshold	0.85
WEC	balance factor	0.8
	edge weight	0.7
	enrichment	0.8
	filtering	0.9

**Table 5:** The evaluation results by different algorithms on five PPI networks.

Network	Criteria	SEGC	CFinder	DPClus	IPCA	Core	SR-MCL	PEWCC	DCU	WCOACH	WEC
Gavin02	Precision	0.5621	0.7333	0.4679	0.4675	0.3717	0.7818	0.5154	0.3897	0.6311	0.7137
	Recall	0.3603	0.1373	0.3088	0.3505	0.3505	0.1838	0.2034	0.2990	0.1520	0.1667
	*F*-measure	**0.4391**	0.2312	0.3721	0.4006	0.3608	0.2977	0.2917	0.3384	0.2449	0.2702
	PPV	0.5597	0.4150	0.6207	0.5541	0.6153	0.5089	0.5558	0.4184	0.3310	0.5936
	Sn	0.4146	0.3203	0.2755	0.3646	0.3646	0.2833	0.2776	0.4490	0.4188	0.2531
	Accuracy	**0.4817**	0.3646	0.4135	0.4495	0.4736	0.3797	0.3928	0.4334	0.3723	0.3876
	Coverage	1352 (**100%**)	623 (46%)	690 (51%)	1352 (100%)	1041 (77%)	584 (43%)	599 (44%)	1350 (100%)	1034 (76%)	502 (37%)
Gavin06	Precision	0.4754 (0.5030)	0.6633	0.5502	0.5289	0.4869	0.7512	0.4687	0.3295	0.4742	0.7774
	Recall	0.3750 (0.4706)	0.1912	0.3873	0.3750	0.3627	0.3088	0.3456	0.2451	0.2328	0.2941
	*F*-measure	0.4193 (**0.4863**)	0.2968	**0.4546**	0.4389	0.4157	0.4377	0.3978	0.2811	0.3123	0.4268
	PPV	0.5335 (0.6110)	0.3425	0.6413	0.5375	0.5833	0.5286	0.5585	0.2959	0.3300	0.5735
	Sn	0.5021 (0.4661)	0.5125	0.4307	0.4807	0.4599	0.4849	0.4307	0.5318	0.5500	0.4479
	Accuracy	0.5176 (**0.5337**)	0.4190	**0.5256**	0.5083	0.5180	0.5063	0.4905	0.3966	0.4261	0.5068
	Coverage	1430 (**100%**)	1124 (79%)	1056 (74%)	1430 (100%)	1144 (80%)	1135 (79%)	1081 (76%)	1413 (99%)	1335 (93%)	947 (66%)
Krogan_core	Precision	0.4889	0.6174	0.3626	0.4732	0.2960	0.7341	0.5379	0.2272	0.5166	0.8382
	Recall	0.5760	0.2034	0.5931	0.5662	0.5907	0.3309	0.3431	0.4779	0.2549	0.2770
	*F*-measure	**0.5289**	0.3060	0.4501	0.5155	0.3943	0.4562	0.4190	0.3080	0.3414	0.4163
	PPV	0.6222	0.3588	0.7128	0.6058	0.6308	0.6063	0.5550	0.3180	0.2231	0.6603
	Sn	0.5885	0.4802	0.4885	0.5786	0.5109	0.4620	0.4135	0.5964	0.5849	0.3937
	Accuracy	**0.6051**	0.4151	0.5901	0.5921	0.5677	0.5293	0.4791	0.4355	0.3612	0.5099
	Coverage	2708 (**100%**)	1143 (42%)	1727 (64%)	2708 (100%)	2082 (77%)	1188 (44%)	1101 (41%)	2660 (98%)	2112 (78%)	866 (32%)
Krogan_extend	Precision	0.4517	0.4545	0.3187	0.4114	0.2036	0.7627	0.4259	0.1450	0.2381	0.7901
	Recall	0.5466	0.1495	0.5711	0.4926	0.5833	0.2794	0.4044	0.4265	0.1789	0.2157
	*F*-measure	**0.4946**	0.2250	0.4091	0.4484	0.3019	0.4090	0.4149	0.2164	0.2043	0.3389
	PPV	0.5564	0.2223	0.6738	0.5234	0.6326	0.5977	0.5179	0.2931	0.1028	0.5935
	Sn	0.6130	0.5625	0.5005	0.5974	0.5125	0.4495	0.4865	0.6271	0.6833	0.3786
	Accuracy	**0.5840**	0.3536	0.5807	0.5592	0.5694	0.5183	0.5019	0.4288	0.2650	0.4740
	Coverage	3672 (**100%**)	1596 (43%)	1948 (53%)	3672 (100%)	2669 (73%)	1282 (35%)	1567 (43%)	3668 (100%)	3309 (90%)	905 (25%)
BioGrid	Precision	0.5377	0.4225	0.3736	0.5075	0.2467	0.5872	0.4923	0.1530	0.1640	0.6600
	Recall	0.8284	0.1520	0.7402	0.8088	0.6667	0.5098	0.7721	0.3113	0.2598	0.4706
	*F*-measure	**0.6521**	0.2235	0.4965	0.6237	0.3602	0.5458	0.6012	0.2051	0.2011	0.5494
	PPV	0.4741	0.1616	0.6031	0.4482	0.5231	0.5019	0.5002	0.2086	0.1530	0.4685
	Sn	0.8104	0.8755	0.6776	0.7885	0.7453	0.7479	0.7344	0.8875	0.9370	0.6922
	Accuracy	0.6199	0.3762	**0.6393**	0.5945	0.6244	0.6127	0.6061	0.4303	0.3786	0.5695
	Coverage	4187 (**100%**)	2740 (65%)	2599 (62%)	4187 (100%)	3243 (80%)	2764 (66%)	2632 (63%)	4168 (99%)	3904 (93%)	2011 (48%)

**Table 6:** Performance of seed-expansion graph clustering (SEGC) on data sets.

Criteria	Gavin02	Gavin06	Krogan_Core	Krogan_Extend	BioGrid
Precision	0.5520 ± 1.1347 × 10^−5^	0.4634 ± 1.5535 × 10^−5^	0.4812 ± 7.0585 × 10^−6^	0.4465 ± 3.9754 × 10^−6^	0.5317 ± 4.8829 × 10^−6^
Recall	0.3603 ± 7.7192 × 10^−30^	0.3708 ± 9.4804 × 10^−6^	0.5727 ± 3.1782 × 10^−6^	0.5425 ± 5.7244 × 10^−6^	0.8257 ± 2.9649 × 10^−6^
*F*-measure	0.4360 ± 1.1076 × 10^−6^	0.4120 ± 7.4338 × 10^−6^	0.5230 ± 2.9506 × 10^−6^	0.4898 ± 2.6329 × 10^−6^	0.6468 ± 3.0660 × 10^−6^
PPV	0.5564 ± 7.5655 × 10^−6^	0.5327 ± 8.7280× 10^−6^	0.6227 ± 1.0720 × 10^−5^	0.5548 ± 2.4239 × 10^−6^	0.4752 ± 2.4318 × 10^−6^
Sn	0.4147 ± 1.9255 × 10^−7^	0.5012 ± 7.4491 × 10^−7^	0.5882 ± 3.4486 × 10^−7^	0.6121 ± 5.7970 × 10^−7^	0.8111 ± 7.6118 × 10^−7^
Accuracy	0.4803 ± 1.7024 × 10^−6^	0.5167 ± 1.9278 × 10^−6^	0.6052 ± 2.5290 × 10^−6^	0.5828 ± 8.2139 × 10^−7^	0.6209 ± 1.1542× 10^−6^

**Table 7:** Examples of predicted complexes by SEGC.

ID	Predicted Complexes	NA	Biological Processes		Molecular Functions		Cellular Components	
			GO Term	*p*-Value	GO Term	*p*-Value	GO Term	*p*-Value
1	YLR370C YIL062C YKL013C YNR035C YJR065C YDL029W YBR234C	1	actin cytoskeleton organization (GO:0030036)	1.59 × 10^−11^	adenyl ribonucleotide binding (GO:0032559)	0.00469	Arp2/3 protein complex (GO:0005885)	9.17 × 10^−22^
2	YBR254C YKR068C YDR472W YDR108W YOR115C YGR166W YDR407C YMR218C YML077W YDR246W	1	Golgi vesicle transport (GO:0048193)	7.60 × 10^−15^	Rab guanyl-nucleotide exchange factor activity (GO:0017112)	9.00 × 10^−20^	TRAPP complex (GO:0030008)	4.05 × 10^−30^
3	YPL007C YBR123C YOR110W YAL001C YGR047C YDR362C	1	transcription from RNA polymerase III type 2 promoter (GO:0001009)	1.91 × 10^−19^	RNA polymerase III type 2 promoter sequence-specific DNA binding (GO:0001003)	1.75 × 10^−19^	transcription factor TFIIIC complex (GO:0000127)	1.01× 10^−19^
4	YJR082C YFL024C YOR244W YNL107W YJL081C YOL012C YFL039C YGR002C YHR090C YHR099W YNL136W YDR359C YEL018W YPR023C YDR485C	0.87	histone acetylation (GO:0016573)	2.67 × 10^−17^	histone acetyltransferase activity (GO:0004402)	2.06 × 10^−13^	NuA4 histone acetyltransferase complex (GO:0035267)	1.26 × 10^−34^
5	YKL139W YJL006C YML112W YAL005C YBR169C	0.6	positive regulation of translational fidelity (GO:0045903)	3.22 × 10^−7^	-	-	carboxy-terminal domain protein kinase complex (GO:0032806)	2.37 × 10^−6^
6	YAL058W YFR042W YPR159W YOR336W YGR143W	-	beta-glucan biosynthetic process (GO:0051274)	3.23 × 10^−9^	glucosidase activity (GO:0015926)	0.00067	integral component of endoplasmic reticulum membrane (GO:0030176)	0.00011
7	YNL263C YGR172C YGL198W YKR014C YML001W YOR089C YNL093W YLR262C YER136W YBR264C YNL044W YER031C YFL038C	-	vesicle-mediated transport (GO:0016192)	1.67 × 10^−11^	GTPase activity (GO:0003924)	2.24 × 10^−13^	cytoplasmic vesicle (GO:0031410)	8.39 × 10^−8^

## References

[1] Mora A., Donaldson I.M. (2011). iRefR: An R package to manipulate the iRefIndex consolidated protein interaction database. BMC Bioinform..

[2] Cao R., Cheng J. (2015). Deciphering the association between gene function and spatial gene-gene interactions in 3D human genome conformation. BMC Genom..

[3] Cao R., Cheng J. (2016). Integrated protein function prediction by mining function associations, sequences, and protein-protein and gene-gene interaction networks. Methods.

[4] Zhao J., Lei X., Wu F.X. (2017). Predicting Protein Complexes in Weighted Dynamic PPI Networks Based on ICSC. Complexity.

[5] Brun C., Herrmann C., Guenoche A. (2004). Clustering proteins from interaction networks for the prediction of cellular functions. BMC Bioinform..

[6] Gavin A.-C., Bosche M., Krause R., Grandi P., Marzioch M., Bauer A., Schultz J., Rick J.M., Michon A.-M., Cruciat C.-M. (2002). Functional organization of the yeast proteome by systematic analysis of protein complexes. Nature.

[7] Lei X., Liang J. (2017). Neighbor affinity-based core-Attachment method to detect protein complexes in dynamic PPI networks. Molecules.

[8] Alberts B. (1998). The cell as a collection of protein machines: Preparing the next generation of molecular biologists. Cell.

[9] Spirin V., Mirny L.A. (2003). Protein complexes and functional modules in molecular networks. Proc. Natl. Acad. Sci. USA.

[10] Wu M., Li X., Kwoh C.-K., Ng S.-K. (2009). A core-attachment based method to detect protein complexes in ppi networks. BMC Bioinform..

[11] Schaeffer S.E. (2007). Graph clustering. Comput. Sci. Rev..

[12] Girvan M., Newman M.E. (2002). Community structure in social and biological networks. Proc. Natl. Acad. Sci. USA.

[13] Pereira J.B., Enright A.J., Ouzounis C.A. (2004). Detection of functional modules from protein interaction networks. Proteins Struct. Funct. Bioinform..

[14] Van Dongen S.M. (2001). Graph Clustering by Flow Simulation. Ph.D. Thesis.

[15] Qin G., Gao L. (2010). Spectral clustering for detecting protein complexes in protein-protein interaction (ppi) networks. Math. Comput. Model..

[16] Bhowmick S.S., Seah B.S. (2016). Clustering and summarizing protein-protein interaction networks: A survey. IEEE Trans. Knowl. Data Eng..

[17] Palla G., Derenyi I., Farkas I., Vicsek T. (2005). Uncovering the overlapping community structure of complex networks in nature and society. Nature.

[18] Adamcsek B., Palla G., Farkas I.J., Derenyi I., Vicsek T. (2006). CFinder: Locating cliques and overlapping modules in biological networks. Bioinformatics.

[19] Liu G., Wong L., Chua H.N. (2009). Complex discovery from weighted PPI networks. Bioinformatics.

[20] Aggarwal C.C., Reddy C.K. (2013). Data Clustering: Algorithms and Applications.

[21] Bader G.D., Hogue C.W. (2003). An automated method for finding molecular complexes in large protein interaction networks. BMC Bioinform..

[22] Altaf-Ul-Amin M., Shinbo Y., Mihara K., Kurokawa K., Kanaya S. (2006). Development and implementation of an algorithm for detection of protein complexes in large interaction networks. BMC Bioinform..

[23] Li M., Chen J.-E., Wang J.-X., Hu B., Chen G. (2008). Modifying the DPClus algorithm for identifying protein complexes based on new topological structures. BMC Bioinform..

[24] Jiang P., Singh M. (2010). SPICi: A fast clustering algorithm for large biological networks. Bioinformatics.

[25] Gavin A.-C., Aloy P., Grandi P., Krause R., Boesche M., Marzioch M., Rau C., Jensen L.J., Bastuck S., Dumpelfeld B. (2006). Proteome survey reveals modularity of the yeast cell machinery. Nature.

[26] Leung H.C., Xiang Q., Yiu S.-M., Chin F.Y. (2009). Predicting protein complexes from PPI data: A core-attachment approach. J. Comput. Biol..

[27] Ma X., Gao L. (2012). Predicting protein complexes in protein interaction networks using a core-attachment algorithm based on graph communicability. Inf. Sci..

[28] Peng W., Wang J., Zhao B., Wang L. (2015). Identification of Protein Complexes Using Weighted PageRank-Nibble Algorithm and Core-Attachment Structure. IEEE/ACM Trans. Comput. Biol. Bioinform..

[29] Chen B., Shi J., Wu F.-X. (2012). Not all protein complexes exhibit dense structures in S. cerevisiae PPI network. Proceedings of Bioinformatics and Biomedicine.

[30] Chen B., Wu F.-X. (2013). Identifying protein complexes based on multiple topological structures in PPI networks. IEEE Trans. Nanobiosci..

[31] Zaki N., Efimov D., Berengueres J. (2013). Protein complex detection using interaction reliability assessment and weighted clustering coefficient. BMC Bioinform..

[32] Zhao B., Wang J., Li M., Wu F.-X., Pan Y. (2014). Detecting protein complexes based on uncertain graph model. IEEE/ACM Trans. Comput. Biol. Bioinform..

[33] Zhang Y., Lin H., Yang Z., Wang J., Li Y., Xu B. (2013). Protein complex prediction in large ontology attributed protein-protein interaction networks. IEEE/ACM Trans. Comput. Biol. Bioinform..

[34] Kouhsar M., Zare-Mirakabad F., Jamali Y. (2015). WCOACH: Protein complex prediction in weighted PPI networks. Genes Genet. Syst..

[35] Keretsu S., Sarmah R. (2016). Weighted edge based clustering to identify protein complexes in protein-protein interaction networks incorporating gene expression profile. Comput. Biol. Chem..

[36] Whang J.J., Gleich D.F., Dhillon I.S. (2016). Overlapping community detection using neighborhood-inflated seed expansion. IEEE Trans. Knowl. Data Eng..

[37] Nepusz T., Yu H., Paccanaro A. (2012). Detecting overlapping protein complexes in protein-protein interaction networks. Nat. Methods.

[38] Lee A.J., Lin M.-C., Hsu C.-M. (2011). Mining Dense Overlapping Subgraphs in weighted protein-protein interaction networks. Biosystems.

[39] Cao B., Luo J., Liang C., Wang S., Song D. (2015). MOEPGA: A novel method to detect protein complexes in yeast protein-protein interaction networks based on Multi-Objective Evolutionary Programming Genetic Algorithm. Comput. Biol. Chem..

[40] Krogan N.J., Cagney G., Yu H., Zhong G., Guo X., Ignatchenko A., Li J., Pu S., Datta N., Tikuisis A.P. (2006). Global landscape of protein complexes in the yeast Saccharomyces cerevisiae. Nature.

[41] Stark C., Breitkreutz B.-J., Reguly T., Boucher L., Breitkreutz A., Tyers M. (2006). BioGRID: A general repository for interaction datasets. Nucl. Acid. Res..

[42] Pu S., Wong J., Turner B., Cho E., Wodak S.J. (2009). Up-to-date catalogues of yeast protein complexes. Nucl. Acid. Res..

[43] Li X., Wu M., Kwoh C.-K., Ng S.-K. (2010). Computational approaches for detecting protein complexes from protein interaction networks: A survey. BMC Genom..

[44] Shih Y.-K., Parthasarathy S. (2012). Identifying functional modules in interaction networks through overlapping Markov clustering. Bioinformatics.

[45] Brohee S., Van Helden J. (2006). Evaluation of clustering algorithms for protein-protein interaction networks. BMC Bioinform..

